# Association of Hypertension With Telomere Length, Considering Non‐Genetic and Genetic Factors, in Middle‐Aged Koreans

**DOI:** 10.1111/jch.70163

**Published:** 2025-10-17

**Authors:** Younghwa Baek, Hyo‐Jeong Ban, Kyoungsik Jeong, Siwoo Lee, Hee‐Jeong Jin

**Affiliations:** ^1^ Korean Medicine (KM) Data Division Korea Institute of Oriental Medicine Daejeon Republic of Korea

**Keywords:** hypertension, non‐genetic factors, polygenic risk score, telomere

## Abstract

Leukocyte telomere length (LTL) has been associated with hypertension. However, this association remains unclear in middle‐aged populations. This study aimed to investigate the association between LTL and hypertension in middle‐aged Koreans, considering genetic and non‐genetic factors. We used baseline data from middle‐aged participants (aged 30–55 years) in the Korean Medicine Daejeon Citizen Cohort. LTL was measured in 1914 participants using quantitative polymerase chain reaction. We calculated the genome‐wide association study‐based polygenic risk score (PRS) for telomere length. Multivariable regression analysis was conducted to examine the association between LTL and hypertension and to explore this association based on non‐genetic and genetic factors. After adjusting most variables (Model 1), individuals in the highest LTL quartile showed an inverse association with hypertension compared to those in the lowest quartile (odds ratio [OR] = 0.60, 95% confidence interval [CI] 0.41–0.86). When further adjusted for antihypertensive medication (Model 2), the association remained but was borderline (OR = 0.66, 95% CI = 0.42–1.04). This inverse association was more clearly observed in stratified subgroups of younger individuals (<45 years), those with optimal low‐density lipoprotein cholesterol levels (<130 mg/dL), and those with adequate sleep duration (≥ 6 h). Hypertension showed a weak association with PRS; there was no significant relationship between PRS and age. Our findings suggest that LTL is independently associated with hypertension in middle‐aged populations; this association varied according to non‐genetic factors. These results demonstrate the potential of using LTL as a measure for hypertension screening and for the development of personalized intervention strategies in healthy populations.

## 1 Introduction

High blood pressure is a major modifiable risk factor for cardiovascular disease (CVD) worldwide. The global prevalence of hypertension in adults is estimated to be 31% [1]; since 1990, the prevalence of hypertension has nearly doubled, making it a significant condition that requires prevention and treatment owing to the risk of hypertension‐related mortality [2]. In Korea, the average blood pressure of adults aged ≥ 20 years is 119/74 mmHg and the prevalence of hypertension is 28%, showing a slight decrease over the past decade [3]. However, owing to the rapidly aging population, the absolute number of patients with hypertension (12 million) and the burden of treatment costs (estimated at 5 trillion KRW annually) have increased [4]. In addition, there is a rising prevalence of hypertension among younger age groups and poor treatment compliance [5]. Therefore, there is an urgent need to identify the risk factors of hypertension for adequate prevention.

Telomeres are repetitive protein‐DNA complex structures located at the ends of chromosomes, and they regulate cellular replicative capacity, prevent chromosomal fusion during cell division, and prevent the loss of genetic data [6]. Telomere length, usually measured in leukocytes, is inversely related to age and considered a marker of biological aging [7]. Telomeric repeats range in size from 0.15 to 50 kilobases and progressively shorten with each cell division [7]; in addition, individual differences in telomere length are affected by non‐genetic and genetic factors. Previous studies have identified various non‐genetic factors, including demographic, social, and environmental factors, that affect leukocyte telomere length (LTL). In particular, lifestyle behaviors such as sleep, smoking, and diet have been shown to influence LTL [8, 9]. Additionally, LTL has been significantly associated with classical cardiovascular risk factors such as blood pressure and cholesterol levels [10, 11]. Regarding genetic factors, several studies have reported that many genes are involved in telomere length maintenance and stability [12, 13].

Multiple epidemiological studies have shown that shorter LTL is associated with hypertension [14, 15, 16]. Mechanistically, age‐dependent telomere dysfunction may contribute to the pathogenesis of hypertension by promoting endothelial and hemodynamic dysfunction [17] and accelerating atherosclerosis [18]. Moreover, long‐term exposure to risk factors such as oxidative stress and chronic inflammation may mediate the association between LTL and hypertension [19]. Meta‐analyses suggest that LTL may be shorter in individuals with hypertension compared to those with normal blood pressure [14]. However, findings across studies have been inconsistent, possibly due to heterogeneity in study design (cross‐sectional vs. longitudinal), population characteristics (e.g., age, ethnicity, comorbidities), sample sizes, and methods of telomere length measurement. Furthermore, most existing studies have focused on older populations or patients with overt CVD, whereas limited attention has been given to middle‐aged individuals—particularly those in the transition to early aging—who may be at a critical stage for early vascular changes and intervention [20]. Therefore, further research is warranted to clarify the relationship between LTL and hypertension in this understudied age group, while accounting for a broad range of influencing factors.

In the present study, we aimed to investigate the relationship between telomere length and hypertension in middle‐aged Koreans, considering genetic and non‐genetic factors that affect telomere length.

## 2 Methods

### 2.1 Study Design and Population

This cross‐sectional study obtained data from the community‐based Korean Medicine Daejeon Citizen Cohort (KDCC), encompassing 2000 participants [21]. The KDCC is an ongoing prospective observational cohort study in Daejeon, South Korea that evaluates the association of risk factors such as individual characteristics, clinical parameters, and lifestyle with cardiometabolic diseases in middle‐aged Koreans. Individuals aged 30–55 years and with no history of cancer and CVDs such as myocardial infarction, angina, and stroke were recruited from 2017 to 2019. The present study analyzed data from 1914 participants, including questionnaire responses, blood pressure values, LTL, and genetic data; missing data were excluded.

### 2.2 Blood Pressure Measurement and Hypertension Definition

Systolic blood pressure (SBP) and diastolic blood pressure (DBP) were measured two times, using an automatic blood pressure cuff (FT‐500R PLUS, Jawon Medical Co., Korea) in the morning. After a rest period of approximately 5–10 min, BP was measured twice in a seated position, with at least 1 min between measurements, under the supervision of trained researchers in an attended setting. The two blood pressure measurements were averaged and used for the analysis. Hypertension was defined as SBP ≥ 140 mmHg and/or DPB ≥ 90 mmHg or the use of antihypertensive medications for blood pressure management [22].

### 2.3 Measurement of LTL

LTL was measured using quantitative polymerase chain reaction (PCR) by calculating the telomere repeat copy number relative to single gene copy number (T/S) ratio [23]. Genomic DNA in leukocytes was extracted from peripheral blood samples using the QIAamp DNA blood mini kit (Qiagen, Hilden, Germany). Purified DNA samples were diluted and quantified using NanoDrop 1000 spectrophotometer (Thermo Fisher Scientific, Wilmington, DE, USA). The ratio of T/S (36B4 gene, which encodes acidic ribosomal phosphoprotein) was determined using iQ Multi‐Color Real‐Time PCR Detection System (Bio‐Rad, Hercules, CA, USA). The final concentrations of the PCR reagents were 10 µL of SYBR Green SuperMix (Bio‐Rad), 20 ng of DNA, 0.4 µL of telomere primers (Bioneer, Daejeon, Republic of Korea), and 0.6 µL of 36B4 primers. The primers used for telomere PCR were 5′‐GGTTTTTGAGGGTGAGGGTGAGGGTGAGGGTGAGGGT‐3′ and 5′‐TCCCGACTATCCCTATCCCTATCCCTATCCCTATCCCTA‐3′. The primers used for 36B4 (single‐copy gene) PCR were 5′‐CAGCAAGTGGGAAGGTGTAATC C‐3′ and 5′‐CCCATTCTATCATCAACGGGTACAA‐3′. The reactions were performed using a telomere and 36B4 primers in the same 96‐well plate, with each plate having a reference DNA sample. A four‐point standard curve was established to transform the cycle threshold into DNA nanograms. To calculate a relative value of LTL for each sample, the amount of telomeric DNA was divided by the amount of 36B4 DNA. In a validity test, the coefficient of variation % values were 4.4% for the intra‐assay and 7.6% for the interassay, based on 34 duplicate samples analyzed in two batches.

LTL values were classified into four groups by quartiles: Q1 (T/S ratio ≤ 0.6), Q2 (0.6 < T/S ratio ≤ 0.91), Q3 (0.91 < T/S ratio ≤ 1.28), and Q4 (T/S ratio > 1.28). The lowest LTL quartile (Q1) had a shorter telomere length and the highest LTL quartile (Q4) had a longer telomere length.

### 2.4 Genotyping and Imputation

DNA samples were quality‐assessed and processed according to standard protocols prior to genotyping using the Precision Medicine Research Array platform from Thermo Fisher Scientific. The platform contains approximately 820 000 single nucleotide polymorphisms (SNPs) optimized for Asian populations [21] according to the Axiom 2.0 Assay Manual Workflow User Guide, the standard protocol.

Imputation analysis was performed using the East Asian (ASN, *n* = 286) reference population from the 1000 Genomes Project Phase 3 dataset [24] to improve genomic coverage of untyped variants. The imputation procedure involved the use of IMPUTE2 software, using genomic coordinates mapped to the GRCh37/hg19 human genome assembly [25]. The final imputation dataset comprised 4 022  016 variants (Digital Content S1) that underwent comprehensive quality assessment. Strict filtering criteria were applied to exclude variants with genotype call rates < 95%, deviations from the Hardy–Weinberg equilibrium (*p* < 1×10^−^⁶), and minor allele frequencies < 1% [26].

### 2.5 Polygenic Risk Score Calculation

We performed genome‐wide association studies (GWASs) under an additive genetic model using PLINK v1.90 [27]. Prior to variant selection, we estimated the SNP‐based heritability of telomere length using the Genome‐Wide Complex Trait Analysis software [28]. We constructed a genetic relationship matrix from quality‐controlled SNPs with minor allele frequencies ≥ 0.01. Thereafter, we performed restricted maximum likelihood analysis to estimate the proportion of phenotypic variance exhibited by all genotyped SNPs, resulting in a heritability estimate of 11% for telomere length. Given our modest sample size (*n* = 2000) and absence of an external replication cohort, we applied a significance threshold of *p* < 1×10^−^⁵ to identify 22 robust genetic variants associated with LTL (Digital Content S2). Leave‐one‐chromosome‐out analysis confirmed that 19 of 22 variants (86.4%) remained consistently associated across different chromosomal exclusions (Digital Content S3). Individual polygenic risk score (PRS) was calculated as follows: PRS_*i* = Σ(*j* = 1 to 22) *β*_*j* × *G*_*ij*, where *β*_*j* is the effect size for SNP *j* from our GWAS analysis, *G*_*ij* is the allele type (0, 1, or 2) for individual *i* at SNP *j*, and the summation includes all 22 significant variants. PRS was computed using the cross‐validated optimal linear unbiased prediction (cvBLUP) algorithm with 10‐fold cross‐validation, in which the dataset was randomly divided into training (90%) and validation (10%) sets, with final accuracy averaged across all folds [29]. Participants were ranked by PRS values and divided into 10 equal decile groups (approximately 200 individuals each). High genetic risk was defined as the top 20% of PRS distribution (9th and 10th deciles) or, alternatively, the top 10% (10th decile alone), whereas low genetic risk was defined as the bottom 20% (first and second deciles) or the bottom 10% (first decile alone). We performed linear regression analysis to explore the relationship between PRS and telomere length, adjusting for age, sex, and body mass index (BMI).

### 2.6 Clinical Parameters

Demographic variables (sex and age), lifestyle factors (sleep duration, alcohol consumption, and smoking status), and medication history of antihypertensive drugs were obtained by trained interviewers using standardized questionnaires [21]. Sleep duration was assessed via self‐reporting of the average sleep hours over the past month. Based on a previous study on the independent association between shortened LTL length and long sleep time, this study excluded individuals with sleep time of 9 h or more from the analysis [30]. Alcohol consumption was computed as the average daily amount of alcohol (g/day) consumed based on the quantity, frequency, and volume of consumption over the past year. Participants were classified into the following alcohol consumption based on sex‐specific criteria for average daily alcohol intake: non‐drinkers, responsible drinkers, and hazardous drinkers [31]. Based on responses to the questions “Have you ever smoked more than 100 cigarettes in your life?” and “Do you currently smoke?”, the participants were classified as non‐smokers (former smokers and non‐smokers) and current smokers.

In addition, physical examination variables included weight, height, and BMI, which was calculated by dividing weight by height squared. Blood samples were collected after overnight fasting, and biochemical parameters such as fasting blood glucose (FBG), total cholesterol (TC), triglycerides (TGs), high‐density lipoprotein cholesterol (HDL‐C), and low‐density lipoprotein cholesterol (LDL‐C), and high‐sensitivity C‐reactive protein (hsCRP) levels were determined.

### 2.7 Statistical Analysis

Data were presented as mean ± standard deviation (SD) for continuous data, and frequency and percentage [*N* (%)] for categorical data according to overall and quartiles of LTL. One‐way analysis of variance or chi‐square test was used to analyze the differences in continuous or categorical variables across LTL quartiles. Pearson correlation analysis was used to assess the relationship between log‐transformed LTL and clinical parameters.

The associations of LTL with continuous outcomes (SBP, DBP) and binary outcome (hypertension prevalence) were examined using univariate and multivariate linear or logistic regression models. LTL was analyzed both as a continuous variable (per one‐unit increment in log‐transformed LTL) and as a categorical variable using the highest LTL quartile (Q4) as the reference. PRS was additionally analyzed as a categorical variable, with the lowest decile as the reference group. The results were presented as beta coefficients (mmHg per log‐transformed LTL) and odds ratio (OR) with the corresponding 95% confidence interval (CI). Logistic regression was employed to adjust for potential confounders and to yield interpretable effect estimates with accompanying measures of uncertainty [32]. Three models were constructed. The crude model included only LTL or PRS. Model 1 adjusted for age, sex, BMI, FBG, TC, TG, HDL‐C, LDL‐C, hsCRP levels, smoking status, alcohol consumption, and sleep duration, to account for demographic and metabolic factors associated with hypertension. Model 2 additionally adjusted for antihypertensive drug use to account for treatment effects on blood pressure. Subgroup analyses were performed using chi‐square tests and multivariable logistic regression, stratifying age (<45 years, ≥45 years), sleep duration (≥6 h, <6 h), and LDL‐C (<130 mg/dL, ≥130 mg/dL), which showed significant correlations with LTL. Additionally, the relationship between PRS and LTL was assessed by comparing mean LTL values across PRS deciles. Multivariable logistic regression was performed to examine the association with hypertension considering the relationship between PRS and age groups, whereas the LDL‐C and sleep groups were not analyzed owing to poor fit of model. All statistical analyses were performed using SAS version 9.4 (SAS Institute Inc., Cary, NC, USA), and *p* < 0.05 indicating statistical significance.

## 3 Results

### 3.1 Characteristics of Participants Stratified by LTL

Table 1 shows the characteristics of all participants stratified by LTL quartiles. Among the 1914 participants, 334 (17.5%) had hypertension. The prevalence of hypertension was highest in the group with the shortest LTL (Q1) at 22.3% and lowest in the group with the longest LTL (Q4) at 13.9%, showing a statistically significant difference between LTL groups (*p* = 0.007). Significant differences in age and antihypertensive drug use across LTL quartile groups were observed. Participants in the shortest LTL group (Q1) were more likely to be older and to use antihypertensive medications compared to those in higher quartiles. The mean age in Q1 was 45.0 ± 6.5 years, significantly higher than in Q2 (43.7 ± 7.0 years), Q3 (43.6 ± 6.8 years), and Q4 (43.3 ± 6.9 years) (*p* < 0.001). Similarly, the prevalence of antihypertensive drug use was highest in Q1 (10.8%) compared to Q2 (5.9%), Q3 (5.4%), and Q4 (5.7%) (*p* = 0.002).

**TABLE 1:** General characteristics of the participants stratified by LTL.

Variables	Total (*n* = 1914)	Quartiles of LTL^†^				
		Q1 (short)	Q2	Q3	Q4 (long)	*p* value
T/S ratio	0.95 ± 0.47	0.37 ± 0.16	0.76 ± 0.09	1.09 ± 0.11	1.58 ± 0.23	<0.001
Sex						
Male	592 (30.9)	143 (29.7)	164 (34.3)	132 (27.6)	153 (32.1)	0.124
Female	1322 (69.1)	338 (70.3)	314 (65.7)	347 (72.4)	323 (67.9)	
Age (years)	43.9 ± 6.8	45.0 ± 6.5^a^	43.7 ± 7.0^b^	43.6 ± 6.8^b^	43.3 ± 6.9^b^	<0.001
< 45	958 (50)	208 (43.2)	247 (51.7)	247 (51.6)	256 (53.8)	0.005
≥ 45	956 (50)	273 (56.8)	231 (48.3)	232 (48.4)	220 (46.2)	
SBP (mmHg)	117.0 ± 15.4	117.9 ± 15.9	117.6 ± 15.5	116.4 ± 15.4	116.2 ± 14.7	0.235
DBP (mmHg)	73.6 ± 12.2	74.0 ± 12.5	73.8 ± 12.3	73.1 ± 12.2	73.3 ± 11.6	0.643
BMI (kg/m^2^)	24.4 ± 3.6	24.4 ± 3.6	24.3 ± 3.8	24.3 ± 3.9	24.4 ± 3.3	0.964
FBG (mg/dL)	84.2 ± 16.3	84.1 ± 12.8	85.1 ± 19.9	83.8 ± 16.5	83.9 ± 15.1	0.578
TC (mg/dL)	197.2 ± 34.8	197.4 ± 34.1	197.0 ± 35.5	194.7 ± 34.3	199.7 ± 35.1	0.179
TG (mg/dL)	132.2 ± 123.8	138.8 ± 162.6	129.6 ± 107.9	127.7 ± 119.5	132.8 ± 94.2	0.525
HDL‐C (mg/dL)	56.8 ± 13.9	57.0 ± 14.3	57.0 ± 13.6	56.6 ± 14.2	56.7 ± 13.4	0.972
LDL‐C (mg/dL)	120.0 ± 32.5	118.5 ± 32.1	119.7 ± 32.2	118.5 ± 32.3	123.2 ± 33.3	0.084
hsCRP (mg/L)	1.25 ± 2.8	1.19 ± 2.36	1.04 ± 1.54	1.62 ± 4.48	1.15 ± 1.77	0.530^c^
Sleep duration (h)	6.65 ± 0.98	6.56 ± 1.06	6.62 ± 0.96	6.68 ± 0.95	6.72 ± 0.96	0.082
Alcohol consumption^‡^	9.6 ± 18.9	10.5 ± 18.5	10.0 ± 21.7	8.6 ± 17.6	9.5 ± 17.5	0.472
Non‐drinker	766 (40)	188 (39.1)	213 (44.6)	180 (37.6)	185 (38.9)	0.086
Responsible drinking	944 (49.3)	233 (48.4)	211 (44.1)	252 (52.6)	248 (52.1)	
Hazardous drinking	204 (10.7)	60 (12.5)	54 (11.3)	47 (9.8)	43 (9)	
Current smoking						
No	1683 (87.9)	425 (88.4)	412 (86.2)	428 (89.4)	418 (87.8)	0.501
Yes	231 (12.1)	56 (11.6)	66 (13.8)	51 (10.7)	58 (12.2)	
Antihypertensive drug						
No	1781 (93.1)	429 (89.2)	450 (94.1)	453 (94.6)	449 (94.3)	0.002
Yes	133 (7)	52 (10.8)	28 (5.9)	26 (5.4)	27 (5.7)	
Hypertension						
No	1580 (82.6)	374 (77.8)	398 (83.3)	398 (83.1)	410 (86.1)	0.007
Yes	334 (17.5)	107 (22.3)	80 (16.7)	81 (16.9)	66 (13.9)	

*Note*: Data are presented as *n* (%) or mean ± standard deviation.

Abbreviations: BMI, body mass index; DBP, diastolic blood pressure; FBG, fasting blood glucose; HDL‐C, high‐density lipoprotein cholesterol; hsCRP, high‐sensitivity C‐reactive protein; LDL‐C, low‐density lipoprotein cholesterol; LTL, leukocyte telomere length; Q, quartiles; SBP, systolic blood pressure; TC, total cholesterol; TG, triglyceride.

^†^ The quartiles of LTL are divided as follows: Q1 (shortest), T/S ratio ≤ 0.6; Q2, 0.6 < T/S ratio ≤ 0.91; Q3, 0.91 < T/S ratio ≤ 1.28; Q4 (longest), T/S ratio > 1.28.

^‡^ g/day of pure alcohol consumption.

^a,b^ Post hoc analysis using Scheffe‘s test, ^c^ *p* value is calculated as log‐transformed hsCRP.

### 3.2 Association Between LTL and Blood Pressure

As shown in Table 2, LTL had significant negative correlations with SBP (*r* = −0.058), DBP (*r* = −0.045), and age (*r* = −0.102) and significant positive correlations with LDL‐C (*r* = 0.047) and sleep duration (*r* = 0.053). As shown in Table 3, in the unadjusted model with LTL as a continuous variable, LTL was significantly associated with SBP (*β* = −1.23, 95% CI = −2.17 to −0.28) and DBP (*β* = −0.75, 95% CI = −1.5 to −0.01). In Model 1, which was mostly adjusted for clinical and lifestyle variables, a marginal association remained between LTL and SBP and DBP. However, in Model 2, further adjusted for antihypertensive drugs, no significant association was observed. In the crude model, logistic regression was performed with LTL as a categorical variable, using the lowest quartile (Q1) as a reference (OR = 1.0). Compared to Q1, the odds of hypertension were 30% lower in Q2, 29% lower in Q3, and 44% lower in Q4, based on the formula (1 − OR) × 100 (Q2: OR = 0.7, 95% CI = 0.51–0.97, *p* = 0.032; Q3: OR = 0.71, 95% CI = 0.52–0.98, *p* = 0.038; Q4: OR = 0.56, 95% CI = 0.4–0.79, *p* = 0.001). In Model 1, the association between LTL as quartiles and hypertension remained statistically significant in the Q2 (OR = 0.69, 95% CI = 0.49–0.98, *p* = 0.041) and Q4 (OR = 0.6, 95% CI = 0.41–0.86, *p* = 0.005) groups compared to Q1. In Model 2, only the Q4 group showed a borderline association with hypertension (OR = 0.66, 95% CI = 0.42–1.04, *p* = 0.071).

**TABLE 2:** Correlation between LTL and clinical parameters.

	LTL^†^	
	*r*	*p* value
Age	−0.102	<0.001
SBP	−0.058	0.011
DBP	−0.045	0.048
BMI	−0.013	0.564
FBG	−0.021	0.364
TC	0.011	0.618
TG	−0.032	0.159
HDL‐C	−0.017	0.467
LDL‐C	0.047	0.041
hsCRP	0.013	0.568
Sleep duration	0.053	0.021
Alcohol consumption	−0.030	0.186

Abbreviations: BMI, body mass index; DBP, diastolic blood pressure; FBG, fasting blood glucose; HDL‐C, high‐density lipoprotein cholesterol; hsCRP, high‐sensitivity C‐reactive protein; LDL‐C, low‐density lipoprotein cholesterol; LTL, leukocyte telomere length; SBP, systolic blood pressure; TC, total cholesterol; TG, triglyceride.

^†^ Log transformed‐LTL.

**TABLE 3:** Associations of LTL with hypertension indicators.

	Crude		Model 1		Model 2	
	*β* (mmHg per log‐LTL)	*p* value	*β* (mmHg per log‐LTL)	*p* value	*β* (mmHg per log‐LTL)	*p* value
SBP ^†^						
LTL	−1.23 (−2.17 to −0.28)	0.011	−0.85 (−1.7 to 0)	0.05	−0.69 (−1.54 to 0.16)	0.11
DBP ^†^						
LTL	−0.75 (−1.5 to −0.01)	0.048	−0.59 (−1.24 to 0.07)	0.08	−0.5 (−1.15 to 0.16)	0.138

	OR (95% CI)	*p* value	OR (95% CI)	*p* value	OR (95% CI)	*p* value
Hypertension ^‡^						
LTL Q1	Reference		Reference		Reference	
LTL Q2	0.7 (0.51–0.97)	0.032	0.69 (0.49–0.98)	0.041	0.87 (0.57–1.33)	0.516
LTL Q3	0.71 (0.52–0.98)	0.038	0.75 (0.53–1.07)	0.109	0.99 (0.64–1.51)	0.946
LTL Q4	0.56 (0.4–0.79)	0.001	0.6 (0.41–0.86)	0.005	0.66 (0.42–1.04)	0.071

*Note*: The crude model is the unadjusted, models adjusted for sex, age, BMI, FBG, TC, TG, HDL‐C, LDL‐C, hsCRP, smoking, alcohol consumption, sleep duration (in Model 1), and antihypertensive drug in addition (in Model 2).

Abbreviations: BMI, body mass index; CI, confidence interval; DBP, diastolic blood pressure; FBG, fasting blood glucose; HDL‐C, high‐density lipoprotein cholesterol; hsCRP, high‐sensitivity C‐reactive protein; LDL‐C, low‐density lipoprotein cholesterol; LTL, leukocyte telomere length; OR, odds ratio; Q, quartiles; SBP, systolic blood pressure; TC, total cholesterol; TG, triglyceride.

^†^ Data are expressed as *β* coefficient (95% CI) per 1 log‐transformed LTL increment.

^‡^ Data are expressed as odds ratio (95% CI), with shortest of LTL quartiles being the reference group.

### 3.3 Subgroup Analysis

**FIGURE 1:**
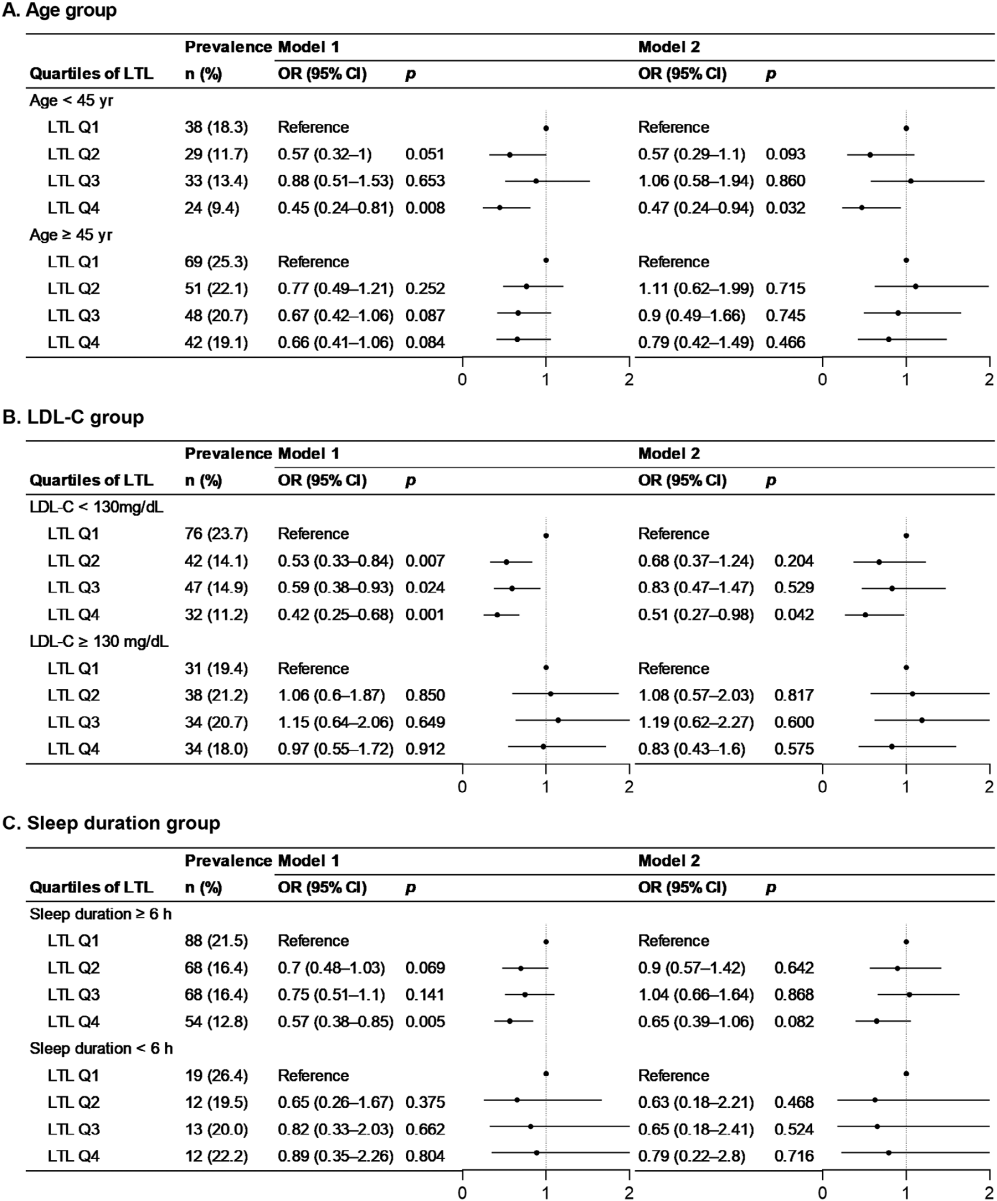
**Association of LTL with hypertension stratified by (A) age, (B) LDL‐C, and (C) sleep duration subgroups**. Odds ratios (ORs) and 95% confidence intervals (CIs) were estimated using logistic regression models. Model 1 was adjusted for sex, age, BMI, glucose, TC, TG, HDL‐C, LDL‐C, hsCRP, smoking, alcohol consumption, and sleep duration. Model 2 further included adjustment for antihypertensive medication use. LTL quartiles were defined as follows: Q1 (T/S ratio ≤ 0.60), Q2 (0.60 < T/S ratio ≤ 0.91), Q3 (0.91 < T/S ratio ≤ 1.28), and Q4 (T/S ratio > 1.28). Q1 represents individuals with the shortest telomere length, whereas Q4 represents those with the longest. ORs represent the odds of having hypertension across LTL quartiles, with Q1 as the reference group. An OR less than 1 indicates a lower risk of hypertension compared to Q1, whereas an OR greater than 1 indicates a higher risk. LDL‐C, low‐density lipoprotein cholesterol; LTL, leukocyte telomere length.

As shown in Figure 1, in the groups with a younger age (< 45 years), lower LDL‐C levels (<130 mg/dL), and sufficient sleep duration (≥ 6 h), the longer LTL group (Q4) had a lower prevalence and odds of hypertension. In younger age group, the Q4 group showed significantly lower odds of hypertension (OR = 0.45, 95% CI = 0.24–0.81, *p* = 0.008) compared to the Q1 group in Model 1; the association was maintained in Model 2 (*p* = 0.032). Among individuals with low LDL‐C levels, hypertension showed a significant association across all long LTL quartiles in Model 1, whereas only the Q4 group maintained a significant relationship with hypertension in the medication‐adjusted Model 2 (*p* = 0.042) (Figure 1B). In participants with sufficient sleep duration, higher LTL was associated with a lower risk of hypertension in Model 1 (OR = 0.57, 95% CI = 0.38–0.85, *p* = 0.005), but this association was not statistically significant in Model 2 (*p* = 0.082) (Figure 1C).

### 3.4 Association Between Genetically Determined Telomere and Hypertension

**FIGURE 2:**
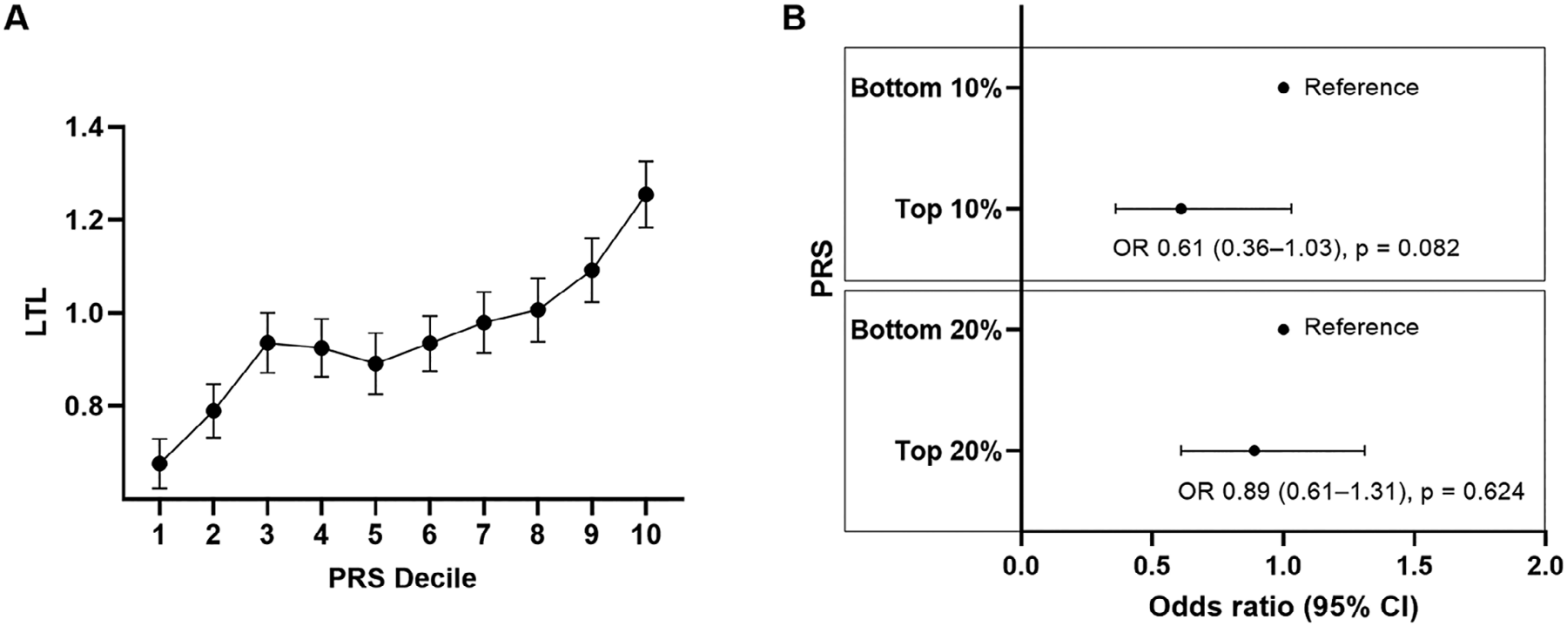
**Associations between polygenic risk score (PRS) for telomere length and (A) mean leukocyte telomere length and (B) odds ratios for hypertension**. A. Mean LTL values were compared across PRS deciles to evaluate the relationship between genetically predicted telomere length and observed LTL. B. Odds ratios (ORs) and 95% confidence intervals (CIs) for hypertension were calculated across PRS groups using logistic regression. PRS values were ranked and divided into decile groups. Bottom 10% (or 20%) refers to genetic risk factors for having short telomere length corresponding to the lower PRS scores. Top 10% (or 20%) refers to genetic risk factors for having longer telomere length corresponding to the higher PRS scores. ORs indicate the relative odds of having hypertension in each PRS group, with the bottom group used as the reference. An OR less than 1 suggests a lower risk of hypertension compared to the reference group, whereas an OR greater than 1 suggests a higher risk. LTL, leukocyte telomere length; PRS, polygenic risk score.

With increasing telomere‐related PRS decile, there was a gradual increase in mean LTL (Figure 2A). There was a 0.6‐fold decrease in the odds ratio of hypertension (OR = 0.61, 95% CI = 0.36–1.03, *p* = 0.082) for individuals with top 10% of PRS compared with individuals in bottom 10% of PRS, showing a borderline association (Figure 2B). When comparing broader risk groups, the top 20% vs. bottom 20% PRS showed a weaker association with hypertension (OR = 0.89, 95% CI = 0.61–1.31, *p* = 0.624). Table 4 presents the PRS‐stratified analysis of the association of age groups with hypertension. In the crude unadjusted analysis, younger age groups (age < 45) showed significantly lower odds of hypertension in both genetic risk groups: 52% lower risk in the high genetic risk group (OR = 0.48, 95% CI = 0.27–0.83, *p* = 0.009) and 58% lower risk in the low genetic risk group (OR = 0.42, 95% CI = 0.23–0.76, *p* = 0.004). After adjusting for covariates in Model 1, younger age groups (age < 45) maintained significantly reduced odds of hypertension with 57% lower risk in the high genetic risk group (OR = 0.43, 95% CI = 0.22–0.81, *p* = 0.009) and a 50% lower risk in the low genetic risk group (OR = 0.5, 95% CI = 0.26–0.94, *p* = 0.031). However, in Model 2 with additional adjustment for antihypertensive medication, these associations were no longer statistically significant in either group (high genetic risk: OR = 0.65, 95% CI = 0.31–1.36, *p* = 0.251; low genetic risk: OR = 0.87, 95% CI = 0.4–1.89, *p* = 0.725). There was no interaction effect between age and genetic risk groups for hypertension (*p* for interaction > 0.05).

**TABLE 4:** The associations of genetic risk and age with hypertension.

	No. of hypertension	Crude		Model 1		Model 2	
	(yes/no)	OR (95% CI)	*p* value	OR (95% CI)	*p* value	OR (95% CI)	*p* value
High genetic risk							
Age ≥ 45	43/154	Reference		Reference		Reference	
Age < 45	22/166	0.48 (0.27–0.83)	0.009	0.43 (0.22–0.81)	0.009	0.65 (0.31–1.36)	0.251
Low genetic risk							
Age ≥ 45	41/158	Reference		Reference		Reference	
Age < 45	18/165	0.42 (0.23–0.76)	0.004	0.5 (0.26–0.94)	0.031	0.87 (0.4–1.89)	0.725
*p* for interaction^†^		0.799		0.83		0.862	

*Note*: High genetic risk refers to the group with a genetic risk for short telomere length in the lower 20% of PRS. Low genetic risk refers to the group with genetic risk for long telomere length in top 20% of PRS.

The crude model is the unadjusted, models adjusted for FBG, TC, TG, HDL‐C, LDL‐C, hsCRP, smoking, alcohol consumption, sleep duration (in Model 1), and antihypertensive drug in addition (in Model 2) using logistic regression.

^†^ Interaction genetic risk group and age group for hypertension.

## 4 Discussion

In the present study, we found a significant inverse association between a long LTL and hypertension in middle‐aged Koreans. Notably, this association varied according to non‐genetic factors such as age, lifestyle, and traditional CVD risk factors. Furthermore, according to GWASs, genetically determined telomere length showed a proportional relationship with LTL measured from blood and a borderline association with hypertension. These findings suggest that telomeres may serve as a potential biological marker for cardiovascular conditions, particularly hypertension, during the early stages of physiological aging in middle adulthood, and that environmental factors may contribute beneficially, influencing the association between telomeres and hypertension.

In our study, individuals in the high LTL group had approximately a 40% lower risk of hypertension compared to those in the low LTL group, and this was associated with reduced SBP and DBP. However, after adjusting for antihypertensive medication, the association between LTL and hypertension was attenuated (Table 3). This finding is consistent with a nationwide survey in Korean adults aged ≥ 18 years, where the inverse association between LTL and hypertension disappeared after adjusting for antihypertensive medication use [15]. Several previous studies have demonstrated an association between shortened LTL and hypertension. The Framingham Heart Study [19] and a pooled meta‐analysis of multiple epidemiological cohorts [14] reported that individuals with hypertension tend to have shorter telomeres [19]. Conversely, in a similar cross‐sectional study involving individuals aged 35–55 years without CVD, the relationship between LTL and hypertension was limited. However, short LTL was associated with unhealthy lifestyle factors and elevated hsCRP levels [20].

Furthermore, in a longitudinal cohort study tracking changes in telomere length among patients with hypertension, a longer LTL was independently associated with reductions in SBP and pulse pressure, particularly among those using antihypertensive medications [16]. Regular antihypertensive treatment may reduce telomerase activity related to telomere shortening, suggesting that long‐term blood pressure control may positively affect telomere preservation [33].

In our study, individuals in the shortest LTL quartile were older on average, with a higher proportion aged ≥ 45 years, consistent with the fact that age is a strong determinant of LTL [34]. To assess whether the association between LTL and hypertension was independent of age, we conducted stratified analyses. Notably, the inverse association was significant only among individuals younger than 45 years, those with LDL‐C levels below 130 mg/dL, and those with adequate sleep duration (≥ 6 h) (Figure 1). Among younger adults, those in the longest LTL quartile had approximately a 50% lower risk of hypertension compared to those in the shortest quartile, even after adjusting for antihypertensive medication. No significant association was observed in older individuals (Figure 1A). These findings suggest that the association between LTL and hypertension is not solely attributable to age. The inverse relationship was evident only in younger adults, where telomere shortening is less advanced, indicating that lifestyle and metabolic factors may also contribute. As telomere attrition is accelerated by aging and modulated by environmental and behavioral influences [35], it may serve as an independent biomarker for hypertension risk, particularly in the early stages of vascular aging in middle‐aged Koreans.

In addition to age, important pathophysiological factors related to dyslipidemia and insufficient sleep contribute to worsening inflammation and oxidative stress, which are not only major causes of CVD but also related to accelerated telomere attrition [33, 36, 37]. In large‐scale, systematically sampled studies, the long LTL group had lower LDL‐C levels than did the short LTL group [15], and dyslipidemia showed a significant inverse correlation with telomere length [38]. Moreover, longitudinal studies revealed that telomere shortening was more pronounced in individuals with persistent dyslipidemia [11] and insufficient or disrupted sleep [36]. Further associations were observed between age, lack of sleep, hypertension, and telomere length [39]. Combined with the results of previous studies, our findings suggest that early intervention on non‐genetic factors in the middle age may enhance the contribution of LTL to overall vascular health.

In the present study, the telomere PRS showed a weak association with hypertension. However, a comprehensive examination of the interaction between age and PRS revealed that the association with hypertension was no longer statistically significant (Table 4 and Figure 2). This suggests that although genetic predisposition may contribute to changes in blood pressure, natural aging, through a process known as telomere shortening, may have a stronger association with hypertension in middle‐aged adults. Also, in the presented study, variants included in the PRS, such as FANCF and TCF12, play important roles in chromosomal stability and DNA repair at the cellular level [40]. Therefore, these genetic variants may affect telomere length.

Genetically longer telomeres are associated with better cardiac function; however, regardless of genetic predisposition, telomere shortening may contribute to cardiovascular dysfunction [10]. In contrast, patients with hypertension had a shorter LTL than did healthy controls, suggesting a possible expression defect in TERT and TERC, the two essential components of the telomerase enzyme complex [41]. Previous study suggested that genetic factors (12.8%) and age (8.5%) were responsible for the largest proportion of variance in LTL [42]. However, in our study, consistency across studies is limited owing to methodological differences, such as study design, sample size, measurement methods, and population characteristics.

This study had several limitations. First, owing to the cross‐sectional design of the study, it was difficult to determine a causal relationship between LTL and hypertension. Second, defining hypertension based on a single‐visit assessment may have led to the inclusion of individuals with white coat hypertension, estimated to affect approximately 15% of the general population [43], potentially leading to an overestimation of its prevalence. Third, despite rigorous adjustments, residual confounding factors may have affected the results. Fourth, the study population was restricted to a specific age group, limiting the generalizability of the findings to other age ranges or ethnic groups. Finally, we applied a relaxed genome‐wide significance threshold (*p* < 1×10^−^⁵) due to our modest sample size (∼2000 participants), which is considerably smaller than those used in large‐scale GWASs. This less‐stringent threshold increases the risk of false positives when analyzing millions of variants. Future studies with longitudinal and mechanistic designs, along with more robust statistical methods (e.g., propensity score matching analysis), are warranted to better elucidate the causal pathways linking LTL shortening to the development of hypertension and to clarify the role of potential modifying factors.

Despite current limitations, our results underscore the preventive value of monitoring telomere length in middle‐aged individuals to identify those at high risk for hypertension and to develop personalized strategies aimed at minimizing LTL shortening through lifestyle modification. Although not yet routine in clinical practice, advances in genomic technologies may soon make LTL monitoring a practical tool for early hypertension risk assessment and prevention.

## 5 Conclusion

In middle‐aged Koreans, a longer LTL was inversely associated with hypertension prevalence, with the association being more significant among younger individuals, those with optimal LDL‐C levels, and those with adequate sleep. Although the study suggests the potential biological role of genetic predisposition in blood pressure regulation, further detailed research is required. This study proposes the potential utility of LTL as a biomarker for hypertension prevention and management and introduces a novel strategy for identifying high‐risk individuals and guiding personalized lifestyle interventions among healthy populations.

## Author Contributions

Conceptualization: Y.B. Data curation and resources: K.J. Formal analysis: Y.B. and H.J.B. Writing—original draft preparation: Y.B. Writing—review and editing: Y.B., H.J.B., and H.J.J. Supervision: H.J.J. and S.L. Funding acquisition: S.L. All authors have read and agreed to the final version of the manuscript.

## Ethics Statement

This study was approved by the Ethics Committee of Korea Institute of Oriental Medicine and Daejeon Korean Medicine Hospital (Approval No: I‐1703/002–002, DJDSKH‐17‐BM‐12, trial registration No. KCT0004297) and was by the Declaration of Helsinki.

## Consent

Written informed consents were obtained from all participants.

## Conflicts of Interest

The authors declare no conflicts of interest.

## Supporting information




**Supplemental Digital Content 1**: Number of single nucleotide polymorphism (SNPs) retained by chromosome after each quality‐control step.
**Supplemental Digital Content 2**: Twenty‐two variants associated with leukocyte telomere length (p < 1x10^−5^).
**Supplemental Digital Content 3**: Leave‐one‐chromosome‐out (LOCO) validation results for significant GWAS variants.

## Data Availability

The datasets generated and analyzed during the current study are not publicly available due to confidentiality and ethical restrictions. However, further inquiries can be directed to the corresponding author.
