# Supplementary material for: Association of Hypertension With Telomere Length, Considering Non‐Genetic and Genetic Factors, in Middle‐Aged Koreans
Source: J Clin Hypertens (Greenwich). 2025 Oct 17;27(10):e70163. doi: 10.1111/jch.70163 (PMC12533746; doi:10.1111/jch.70163)
Supplement: Supplementary file 1 — Supplemental Digital Content 1: Number of single nucleotide polymorphism (SNPs) retained by chromosome after each quality‐control step. Supplemental Digital Content 2: Twenty‐two variants associated with leukocyte telomere length (p < 1x10^−5^). Supplemental Digital Content 3: Leave‐one‐chromosome‐out (LOCO) validation results for significant GWAS variants. [file JCH-27-e70163-s001.docx]

**[Supplemental Digital Contents]**

**Supplemental Digital Content 1. Number of single nucleotide polymorphism (SNPs) retained by chromosome after each quality-control step**

| **Chromosome** | **All Variants** | **GENO Filter** | **MAF Filter** | **HWE Filter** | **Filtered Variants** |
| --- | --- | --- | --- | --- | --- |
| chr1 | 819,930 | 612,894 | 317,579 | 317,575 | 317,575 |
| chr2 | 827,171 | 625,194 | 333,701 | 333,699 | 333,699 |
| chr3 | 810,927 | 623,703 | 296,838 | 296,838 | 296,838 |
| chr4 | 699,820 | 524,428 | 293,594 | 293,591 | 293,591 |
| chr5 | 625,331 | 470,243 | 256,893 | 256,893 | 256,893 |
| chr6 | 728,736 | 555,839 | 292,089 | 292,085 | 292,085 |
| chr7 | 588,421 | 424,784 | 229,029 | 229,028 | 229,028 |
| chr8 | 528,362 | 394,645 | 215,639 | 215,638 | 215,638 |
| chr9 | 496,167 | 365,699 | 172,436 | 172,436 | 172,436 |
| chr10 | 501,947 | 380,658 | 208,895 | 208,895 | 208,895 |
| chr11 | 473,696 | 359,415 | 199,456 | 199,455 | 199,455 |
| chr12 | 527,056 | 400,463 | 196,275 | 196,274 | 196,274 |
| chr13 | 403,164 | 306,054 | 148,837 | 148,837 | 148,837 |
| chr14 | 374,612 | 282,133 | 132,515 | 132,515 | 132,515 |
| chr15 | 331,879 | 240,728 | 114,975 | 114,970 | 114,970 |
| chr16 | 364,694 | 260,595 | 115,056 | 115,053 | 115,053 |
| chr17 | 311,766 | 230,456 | 103,857 | 103,855 | 103,855 |
| chr18 | 324,336 | 243,760 | 115,542 | 115,542 | 115,542 |
| chr19 | 244,440 | 174,727 | 84,905 | 84,905 | 84,905 |
| chr20 | 253,680 | 188,982 | 87,190 | 87,189 | 87,189 |
| chr21 | 161,676 | 116,332 | 54,724 | 54,724 | 54,724 |
| chr22 | 145,583 | 101,803 | 52,025 | 52,019 | 52,019 |
| Total | 10,543,394 | 7,883,535 | 4,022,050 | 4,022,016 | 4,022,016 |

SNP, single nucleotide polymorphism; GENO, genotype call rate; MAF, minor allele frequency; HWE, Hardy-Weinberg equilibrium **Supplemental Digital Content 2. Twenty-two variants associated with leukocyte telomere length (P < 1x10^-5^)**

| **SNP**  **(#Chromosome)** | **BP** | **Gene** | **A1** | **A2** | **EAF** | **Effect** | **SE** | **P value** | **N** | **P(HET)** |
| --- | --- | --- | --- | --- | --- | --- | --- | --- | --- | --- |
| AX-86739449 (#1) | 70671219 | LRRC40 | T | G | 0.0288 | 0.2362 | 0.0462 | 3.44E-07 | 1913 | 0.4089 |
| rs200270236 (#2) | 113486136 | NT5DC4 | A | C | 0.015 | -0.2897 | 0.0626 | 4.02E-06 | 1913 | 1 |
| rs1456140 (#3) | 150712861 | CLRN1-AS1 | T | C | 0.3974 | -0.0713 | 0.0155 | 4.76E-06 | 1891 | 1 |
| rs6763580 (#3) | 150714425 | CLRN1-AS1 | A | G | 0.4098 | -0.0693 | 0.0153 | 6.48E-06 | 1913 | 0.9263 |
| rs56281950 (#3) | 150716019 | CLRN1-AS1 | G | T | 0.3599 | 0.0734 | 0.0160 | 4.56E-06 | 1877 | 0.6592 |
| rs140718662 (#4) | 76877285 | SDAD1 | G | C | 0.0278 | 0.2494 | 0.0467 | 1.06E-07 | 1913 | 0.4024 |
| rs4728190 (#7) | 129855837 | SSMEM1 | T | C | 0.0263 | 0.2444 | 0.0481 | 4.08E-07 | 1913 | 0.6456 |
| rs7900846 (#10) | 94901328 | XRCC6P1 | T | C | 0.0931 | -0.1143 | 0.0257 | 9.04E-06 | 1894 | 0.2291 |
| rs7915548 (#10) | 94901352 | intergenic variant | A | G | 0.1094 | -0.1054 | 0.0236 | 8.81E-06 | 1896 | 0.06436 |
| rs730880277 (#11) | 22647105 | FANCF | - | CTGGAAGTTCGCTAATCCCGGAA | 0.0413 | -0.1792 | 0.0389 | 4.33E-06 | 1913 | 0.04696 |
| rs371886513 (#11) | 65661565 | FOSL1 | A | G | 0.0293 | 0.2320 | 0.0458 | 4.41E-07 | 1913 | 0.4136 |
| rs144192164 (#12) | 112184437 | ACAD10 | T | C | 0.0167 | 0.2621 | 0.0592 | 9.99E-06 | 1890 | 1 |
| rs2733187 (#15) | 57370220 | TCF12 | A | G | 0.1769 | 0.0892 | 0.0198 | 7.37E-06 | 1832 | 0.2095 |
| rs2703588 (#15) | 57376765 | TCF12 | G | A | 0.1654 | 0.0894 | 0.0201 | 9.44E-06 | 1881 | 0.3268 |
| rs72731964 (#15) | 57452024 | TCF12 | T | A | 0.1715 | 0.0876 | 0.0197 | 8.71E-06 | 1910 | 0.2072 |
| rs3794617 (#15) | 57458784 | TCF12 | T | C | 0.1716 | 0.0873 | 0.0196 | 9.50E-06 | 1909 | 0.2073 |
| rs72731974 (#15) | 57462177 | TCF12 | T | C | 0.1716 | 0.0873 | 0.0196 | 9.50E-06 | 1909 | 0.2073 |
| rs7182188 (#15) | 57503164 | TCF12 | G | T | 0.1688 | 0.0879 | 0.0197 | 9.03E-06 | 1898 | 0.09327 |
| rs146278982 (#15) | 100935938 | CERS3-AS1 | T | C | 0.0618 | 0.1422 | 0.0316 | 7.14E-06 | 1913 | 0.4375 |
| rs142917638 (#17) | 41055975 | G6PC1 | A | G | 0.0225 | -0.2347 | 0.0511 | 4.72E-06 | 1913 | 0.6237 |
| rs1854931 (#20) | 49312771 | LINC01270 | A | G | 0.0208 | 0.2503 | 0.0533 | 2.88E-06 | 1842 | 0.5677 |
| rs190274526 (#22) | 25496226 | KIAA1671 | C | T | 0.0118 | 0.3105 | 0.0695 | 8.59E-06 | 1913 | 1 |

SNP, single nucleotide polymorphism; BP, base pair position; EAF, effect allele frequency; SE, standard error; P(HET), p value (heterogeneous across sexes)

**Supplemental Digital Content 3. Leave-one-chromosome-out (LOCO) validation results for significant GWAS variants**

| **Chr** | **SNP** | **BP** | **EAF** | **Effect** | **SE** | **P value** |
| --- | --- | --- | --- | --- | --- | --- |
| 1 | AX-86739449 | 70671219 | 0.028228 | 0.233947 | 0.046378 | 4.55E-07 |
| 2 | rs200270236 | 113486136 | 0.014898 | -0.29146 | 0.063214 | 4.01E-06 |
| 3 | rs1456140 | 150712861 | 0.398202 | -0.06923 | 0.015588 | 8.95E-06 |
| 3 | rs56281950 | 150716019 | 0.362014 | 0.072314 | 0.015997 | 6.17E-06 |
| 4 | rs140718662 | 76877285 | 0.027444 | 0.247403 | 0.047 | 1.41E-07 |
| 7 | rs4728190 | 129855837 | 0.025876 | 0.241065 | 0.048303 | 6.02E-07 |
| 10 | rs7900846 | 94901328 | 0.092661 | -0.11437 | 0.025816 | 9.42E-06 |
| 10 | rs7915548 | 94901352 | 0.109441 | -0.10562 | 0.023806 | 9.13E-06 |
| 11 | rs730880277 | 22647105 | 0.041296 | -0.174 | 0.039139 | 8.76E-06 |
| 11 | AX-90059872 | 65661565 | 0.028751 | 0.231936 | 0.045929 | 4.42E-07 |
| 12 | 12-112184437 | 112184437 | 0.016931 | 0.265421 | 0.059639 | 8.57E-06 |
| 15 | rs2733187 | 57370220 | 0.179858 | 0.089202 | 0.019935 | 7.66E-06 |
| 15 | 15-57452024 | 57452024 | 0.174084 | 0.087954 | 0.019765 | 8.58E-06 |
| 15 | rs3794617 | 57458784 | 0.174175 | 0.087563 | 0.019767 | 9.44E-06 |
| 15 | 15-57462177 | 57462177 | 0.174175 | 0.087563 | 0.019767 | 9.44E-06 |
| 15 | rs7182188 | 57503164 | 0.171233 | 0.088088 | 0.019875 | 9.33E-06 |
| 17 | rs142917638 | 41055975 | 0.022739 | -0.2286 | 0.051455 | 8.88E-06 |
| 20 | rs1854931 | 49312771 | 0.021173 | 0.249766 | 0.053561 | 3.11E-06 |
| 22 | rs190274526 | 25496226 | 0.012023 | 0.310422 | 0.069695 | 8.43E-06 |

SNP, single nucleotide polymorphism; BP, base pair position; EAF, effect allele frequency; SE, standard error
